# Changes in Parasite Virulence Induced by the Disruption of a Single Member of the 235 kDa Rhoptry Protein Multigene Family of *Plasmodium yoelii*

**DOI:** 10.1371/journal.pone.0020170

**Published:** 2011-05-20

**Authors:** Devaki Bapat, Ximei Huang, Karthigayan Gunalan, Peter R. Preiser

**Affiliations:** School of Biological Sciences, Nanyang Technological University, Singapore, Singapore; Instituto Butantan, Brazil

## Abstract

Invasion of the erythrocyte by the merozoites of the malaria parasite is a complex process involving a range of receptor-ligand interactions. Two protein families termed Erythrocyte Binding Like (EBL) proteins and Reticulocyte Binding Protein Homologues (RH) play an important role in host cell recognition by the merozoite. In the rodent malaria parasite, *Plasmodium yoelii*, the 235 kDa rhoptry proteins (Py235) are coded for by a multigene family and are members of the RH. In *P. yoelii* Py235 as well as a single member of EBL have been shown to be key mediators of virulence enabling the parasite to invade a wider range of host erythrocytes. One member of Py235, *PY01365* is most abundantly transcribed in parasite populations and the protein specifically binds to erythrocytes and is recognized by the protective monoclonal antibody 25.77, suggesting a key role of this particular member in virulence. Recent studies have indicated that overall levels of Py235 expression are essential for parasite virulence. Here we show that disruption of *PY01365* in the virulent YM line directly impacts parasite virulence. Furthermore the disruption of *PY01365* leads to a reduction in the number of schizonts that express members of Py235 that react specifically with the mcAb 25.77. Erythrocyte binding assays show reduced binding of Py235 to red blood cells in the *PY01365* knockout parasite as compared to YM. While our results identify *PY01365* as a mediator of parasite virulence, they also confirm that other members of Py235 are able to substitute for *PY01365*.

## Introduction

Invasion of the red blood cell (rbc) and its subsequent destruction is a main contributor to malaria associated pathology. The mechanism by which the invasive form of the malaria parasites, the merozoite, selects and successfully invades a red blood cell is a complex process involving numerous receptor ligand interactions (reviewed in [1], [2], [3], [4]). The human parasite *Plasmodium falciparum* is able to invade rbc of all ages while *P. vivax* is only able to invade a relatively small subset of circulating rbc, the reticulocytes. Generally, this leads to a significantly lower overall parasite burden in *P. vivax* as compared to *P. falciparum*, resulting in differences in pathology due to parasitaemia. The ability of merozoites to efficiently invade a wider range of rbc is therefore directly linked to pathology with parasites that are able to invade a larger subset of rbc causing more severe disease [5]. Merozoite invasion efficiency and its relationship to pathology are difficult to study in human malaria parasites, making the rodent malaria parasite *P. yoelii* an ideal model. In *P. yoelii* the virulent YM strain is able to invade rbc of all ages [6] while the avirulent 17X1.1 and YA strains are mainly restricted to young erythrocytes [7], reflecting the invasion characteristics of *P. falciparum* and *P. vivax,* respectively. Comparisons of virulent and avirulent clones of *P. yoelii* have identified two protein families, Py235 (*Plasmodium yoelii* 235 kDa rhoptry protein family) and PyEBL (*P. yoelii* Erythrocyte Binding Like) as key mediators of invasion efficiency [8], [9], [10], [11], [12].

Both Py235 as well as PyEBL belong to two gene families conserved in *Plasmodia*, the Reticulocyte Binding Protein homologues (RH) and the Erythrocyte Binding Like (EBL) protein families (reviewed in [1], [2], [3], [13]). The number of members of RH and EBL vary in different parasite species, with *P. falciparum* having 6 members of RH and 5 members of EBL [1] as compared to the approximately 14 RH and two EBL members identified in *P. yoelii* [14], [15], [16], [17]. Variations in the expression of either EBL or RH are linked to changes in the rbc receptors utilized by *P. falciparum* [18], [19], [20], [21], [22], [23], [24], [25], [26], [27], [28], [29], [30], [31], [32], [33], [34], [35], [36]. In *P. yoelii,* passive transfer of monoclonal antibodies targeting Py235 or direct immunization with full length Py235 is able to protect experimental mice from a challenge with the normally lethal YM strain [8], [9], [37], by converting the normally fulminating YM infection to a more reticulocyte restricted infection similar to that observed in infections with the avirulent YA and 17X1.1 strains. In addition merozoites originating from a single schizont transcribe different members of *Py235* suggesting that Py235 is not only a key virulence factor but also a potential mediator of adaptation and immune evasion [38], [39]. Recently, it was shown that Py235 mediated virulence appears not to be due to differences in the *py235* repertoire found in virulent and avirulent lines of *P. yoelii* [15], [40] but rather is a result of the overall upregulation of Py235 expression [27]. In addition to the increased expression of Py235, a single point mutation that leads to the miss-localization of PyEBL from an apical location to the dense granules, has recently been identified as being important for virulence [11], [12]. How the aberrant location of PyEBL can lead to increased invasion and virulence is not clear, though the authors of this study have suggested that it may free up space at the apical end, enabling more proteins like Py235 to be translocated to this functionally important position.

Not all members of Py235 are transcribed at the same level in the mouse with some members making up to 40% of total transcripts while other members are not being transcribed at all [27]. Interestingly, the overall transcriptional relationship of different *py235* is generally conserved in both virulent as well as avirulent parasite lines [27]. In both virulent and avirulent parasites, *PY01365* is the most abundantly transcribed Py235 member [27] and has been shown to be recognized by the protective monoclonal antibody 25.77 [41] suggesting a key role of this particular Py235 in virulence.

In this work we have expanded our understanding on the role of Py235 in parasite mediated virulence. We show that disruption of *PY01365* in the virulent YM line directly impacts parasite virulence and leads to a reduction in the number of schizonts that express members of Py235 that are recognized by the mcAb 25.77. Moreover, we show that disruption of *PY01365* can lead to the upregulation of different *py235* members and that differences in the *py235* transcription pattern can impact on the invasion properties of the parasite. While our results identify PY01365 as one contributor of parasite virulence, it is clear from our data that other members of Py235 can partly compensate for the loss of this gene.

## Results

### Disruption of the most abundantly transcribed member of the *Py235* multigene family

**Figure 1:**
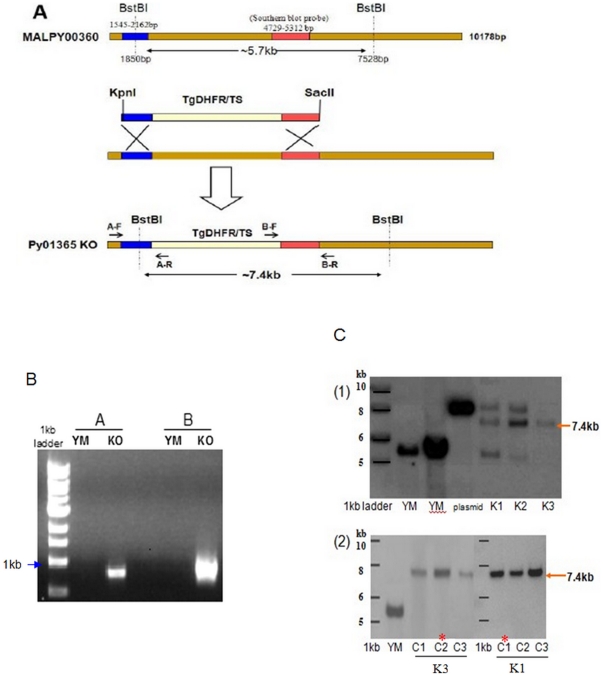
Disruption of *py01365* using homologous recombination. A- genomic locus MALPY00360 coding for *py01365* showing the two regions (blue and red) used for targeting this locus by a double cross-over strategy. Homologous recombination with the linearized plasmid containing the selectable marker flanked by the targeting sequences results in the Py01365 KO locus. Restriction sites used for Southern blot analysis as well as the location of the primer pairs A-F, A-R and B-F, B-R important for PCR screening of both the 5′ and 3′ integration event as well as region used for Southern blot probe are also indicated. B- PCR screening of 5′ (A) and 3′ (B) integration events in both wild type (YM) and knock out (KO) parasites using primers A-F, A-R and B-F, B-R respectively. Both primer pairs are only expected to give a product if integration has occurred. C- Southern blot screening of parasites for correct integration. (1) BstBI digested DNA obtained from wild type (YM) as well as transfected parasites (K1, K2 and K3) and the transfection plasmid (Pl) was analyzed by Southern blot using a *PY01365* specific probe (region indicated in red). The expected fragment of ∼7.4 kb can be seen in all three transfected parasite lines. (2) Transfected clone K1 and K3 were subsequently cloned out by limiting dilution and again screened by Southern blot. Single parasite clone K1-C1 and K3-C2 were selected for further analysis, and were renamed *PYΔpy01365(NF1*)and *PYΔpy01365(NF2*), respectively.

Previous studies using both quantitative RT-PCR or proteomic analysis have identified *PY01365* as a dominant member of Py235 expressed in the virulent *P. yoelii* YM line [27], [41]. A replacement targeting plasmid (Fig. 1) was made to delete most of the open reading frame of *PY01365* by double cross over homologous recombination. Successful integration of the plasmid into the *PY01365* locus can be detected by Southern blot analysis of BstBI digested DNA, with a 7.4 kb band indicating correct integration while a fragment of 5.7 kb represents the original locus. The targeting vector that has not integrated would be detected as an approximately 9 kb band on the blot. Three independent transfections were carried out in *P. yoelii* YM parasites and transfected parasites were initially screened by PCR (Fig. 1B and data not shown) and southern blot (Fig. 1C (1)). Two of the initial transfected parasite populations (Fig. 1C (1), clone K1& K2) in addition to the expected 7.4 kb band also showed bands consistent with residual episomal plasmid as well as intact *PY01365,* while one only had the expected band (Fig. 1C (1), clone K3). Transfected parasite populations K1 and K3 that showed the expected PCR products for both correct 3′ and 5′ integration as well as the expected size fragments by southern blot were subsequently cloned by limiting dilution. Three single-parasite-clones obtained from the two independent transfections were then analyzed by Southern blot for correct integration (Fig. 1C (2)). Clone K1-C1 was renamed as *PYΔpy01365*(NF1), while clone K3-C2 was renamed as *PYΔpy01365*(NF2). The two confirmed *PY01365* knock out parasite were used for all subsequent analysis.

### *PYΔpy01365* affects host survival in BALB/c mice

**Figure 2:**
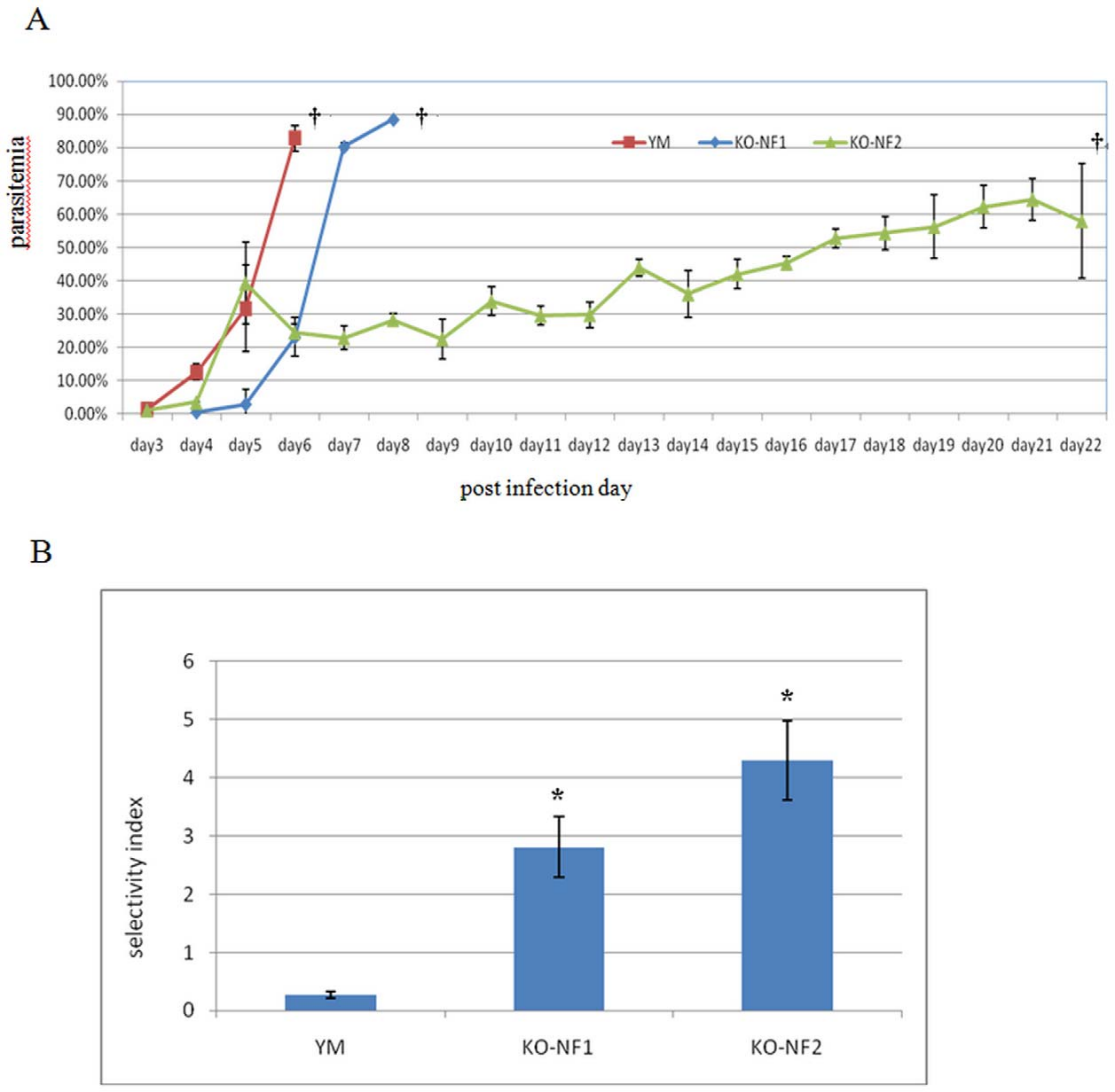
Comparison of growth behavior of YM and *PYΔpy01365.* A- Parasitaemia of BALB/c mice infected with 10^4^ parasites on day 0 was taken daily. The average parasitaemia of 5 mice for both YM and *PYΔpy01365* is represented. Error bars are given for each time point. **†** Indicates death of animals. B- Average Selective index of 5 BALB/c mice infected with either YM or *PYΔpy01365.* Parasites smears were analyzed when parasitaemia was in the range of 5–15%. Differences in SI between YM and *PYΔpy01365*were significant (p<0.01).

To assess the impact of the disruption of *PY01365* on parasite virulence 5 mice were infected with 10^4^ parasites of the virulent YM line, *PYΔpy01365*(NF1) and *PYΔpy01365*(NF2), respectively. The parasitaemia was measured each day after the infection (Fig. 2A). In the virulent YM line parasitaemia rose rapidly and all mice died by day 6 with a peak parasitaemia of >80%. In the *PYΔpy01365*(NF2) clone parasite replication was delayed early in the infection with parasitaemia on day 4 being 3.6% as compared to 12.6% in the YM line (p = 0.0052), subsequently the parasitaemia rose rapidly to peak at around 40% at day 5, the overall parasite load then dropped to as low as 22% at day 9 before gradually rising to another peak of around 65% by day 21. All mice died on day 22 with severe anemia (Fig. 2A). A similar delay in early parasite replication was also seen in *PYΔpy01365*(NF1) were parasitaemia was about 0.5% on day 4 (p = 0.0087) and 2.9% on day 5, compared to 12.6% and 31.71% respectively in the YM line. In *PYΔpy01365*(NF1) the parasitaemia then continued to increase rapidly and all mice died on day 9.

We have previously shown that reduced parasite virulence is linked to an increase in the selectivity index (SI) of the parasite [3], with a higher SI indicating a restricted host cell range. The SI was therefore determined for all the mice at a parasitaemia of between 5–15% (Fig. 2B). There is a significant difference in the average SI in YM ∼0.2 compared to ∼4.2 in *PYΔpy01365*(NF2) (p = 0.0038) and ∼2.8 in *PYΔpy01365*(NF1) (p = 0.001). The SI values observed in this study are similar to the values measured in previous studies where SI values of 0.2 are linked with virulent parasites while SI values of 4 are seen in parasites considered avirulent [3].

### Reduced recognition of schizonts by protective monoclonal antibody 25.77

**Figure 3:**
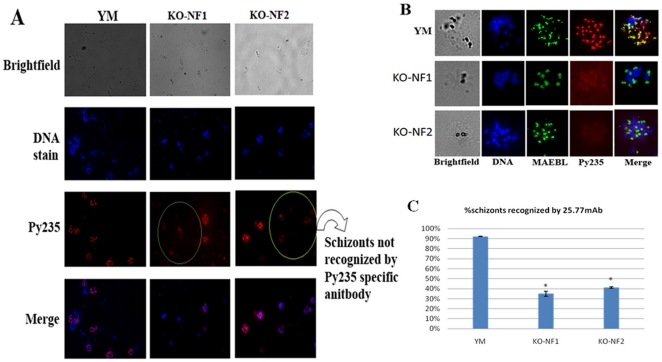
Differences in expression of Py235 recognized by 25.77 in YM or *PYΔpy0136* parasites. A- Immunofluorescence Assays of YM and *PYΔpy01365(NF1*) and *PYΔpy01365(NF2*) with the protective monoclonal antibody 25.77 (Py235). Fewer schizonts in *PYΔpy01365* reacted specifically with the Py235 specific antibody (circled). The specific antibodies that reacted with the schizonts were detected with Alexa Fluor labeled anti-mouse IgG. The fluorescent images (individual stains and merged) and the bright-field are shown. B- Immunofluorescence Assays of YM and *PYΔpy01365* parasites with mcAb 25.77 (Py235) and a rabbit serum against the rhoptry protein MAEBL. The specific antibodies that reacted with the schizonts were detected with Alexa Fluor labeled goat anti rabbit (or anti-mouse) IgG. The fluorescent images (individual stains and merged) and the bright-field are shown. C- Quantification of the number of schizonts that are MAEBL and Py235 positive. YM and *PYΔpy01365* parasites were stained with mcAb 25.77 and a rabbit serum against the rhoptry protein MAEBL. A total of 200 MAEBL positive schizonts were counted and their mcAb 25.77 staining was determined. Comparison of double labeled parasites showed a significant difference between YM and *PYΔpy01365* parasites (p< 0.01).

The Py235 specific monoclonal antibody 25.77 [8] can protect mice against the virulent YM line and has recently been shown to immunoprecipitate PY01365 [41]. To assess whether the disruption of *PY01365* leads to a decrease in the number of schizonts being recognized by 25.77 immunofluorescence assays using fixed YM and *PYΔpy01365*(NF1) and *PYΔpy01365*(NF2) parasites were performed. Nearly all YM parasites are specifically stained by the 25.77 antibody while there is a significant decrease in the number of schizonts that are stained in both the *PYΔpy01365* NF1 and NF2 parasite (Fig. 3A).

To confirm these results schizonts were stained with an antibody (2T8) against the rhoptry protein MAEBL [42] as well as 25.77. In the YM line consistently all schizonts were co-stained by both antibodies while in *PYΔpy01365* NF1 and NF2 a number of parasites only showed staining with the MAEBL specific antibody (Fig. 3B).

To quantify the number of parasites that did not get recognized by 25.77 a total of 200 schizonts that were positive for MAEBL were counted for co-staining with 25.77 in both YM and *PYΔpy01365* NF1 and NF2. In YM an average of ∼90% of schizonts was stained by both antibodies compared to only ∼35% (*p* <0.01) in *PYΔpy01365* NF1 and ∼41% in NF2 (*p* <0.01) (Fig. 3C), suggesting that the disruption of *PY01365* leads to the increased expression of members of Py235 that are not recognized by 25.77. At the same time the results also confirm that the protective 25.77 antibody does not only recognize a single variant of Py235.

### Disruption of *PY01365* leads to a changes in expression of individual *py235*

**Figure 4:**
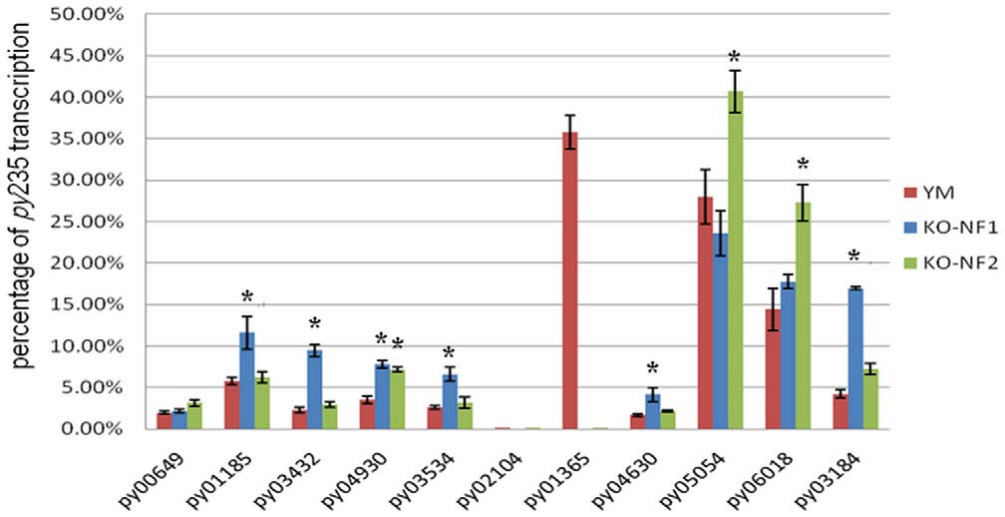
Transcription of *py235* in YM and *PYΔpy01365* parasites. Analysis of changes of the transcription pattern of different *py235* members by quantitative reverse transcription - real time-PCR. Analysis of transcription levels of 11 different *py235* members in YM (red) and *PYΔpy01365(NF1*) (blue) and *PYΔpy01365(NF2*) (green). Results are expressed as percent of total *py235* transcription. * indicates statistically significant differences in the transcription levels of a gene between YM and *PYΔpy01365* parasites (p<0.05).

To assess whether disruption of *PY01365* leads to a change in the overall transcription pattern of different members of *Py235* the transcription levels of each *Py235* gene was assessed by quantitative RT-PCR. This analysis clearly shows that there is an overall change in relative transcription of different *py235* in the knockout parasites as compared to YM. In *PYΔpy01365(NF1*) there is a significant (p<0.05) increase in the contribution of, *PY03432, PY04930, PY03534,* and *PY03184*, while the levels of *PY00649, PY01185, PY04630, PY05054* and *PY06018* show no significant change as compared to YM (Fig. 4). In contrast in *PYΔpy01365(NF2*) *PY04930, PY05054, PY06018* and *PY03184* levels are significantly (p<0.05) increased while the levels of *PY01185, PY03432, PY00649, PY04630* and *PY03534* remain unchanged. It is interesting to note that only *PY04930* showed a similar increase in transcription in both NF1 and NF2, while only *PY00649* remained unchanged in both the knockout parasites. Importantly, no *PY01365* transcript was detected in the *PYΔpy01365(NF1*) and *PYΔpy01365(NF2*) parasite clones confirming the genetic disruption of this gene.

### Reduced erythrocyte binding of Py235 in *PYΔpy01365(NF2*)

**Figure 5:**
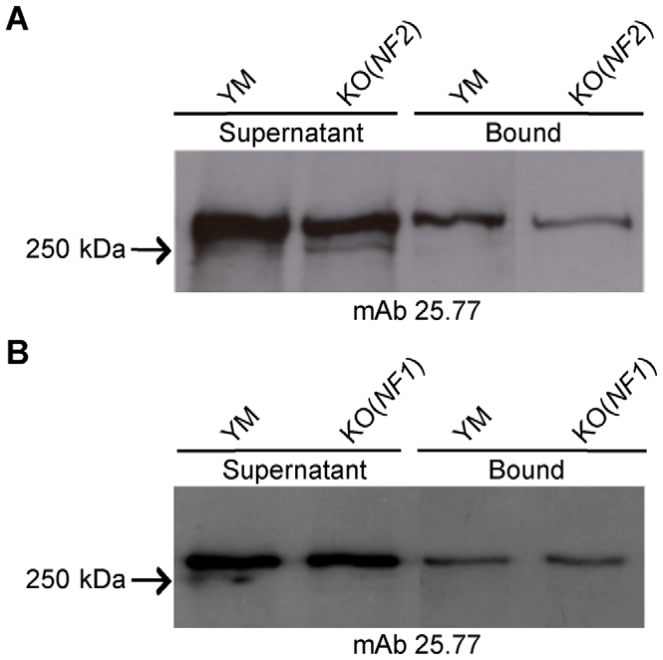
Erythrocyte binding assay of parasite culture supernatant from both YM and *PYΔpy01365*. Western blot analysis using mcAb 25.77 of equal amounts of parasite culture supernatant as well as proteins bound to erythrocytes from A) YM and *PYΔpy01365(NF2*) as well as B) YM and *PYΔpy01365(NF1*).

Since mice infected with *PYΔpy01365(NF2*) survived significantly longer than those infected with *PYΔpy01365(NF1*) it was important to establish whether there were any differences in the ability of the other members of Py235 to bind to red blood cells. Western blot analysis of parasite culture supernatant [43], [44] from both YM and *PYΔpy01365* using mcAb 25.77 clearly detected Py235 in both parasite clones (Fig. 5). Erythrocyte Binding Assays (EBA) carried out using equal amounts of parasite culture supernatant [43], [44] showed binding of Py235 from both YM and *PYΔpy01365(NF2*) (Fig. 5A) as well as YM and *PYΔpy01365(NF1*) (Fig. 5B). Quantification of total Py235 detected in the supernatant as well as bound to erythrocytes showed that approximately 36% of the mcAb 25.77 reactive Py235 from the YM and 12% from the *PYΔpy01365(NF2)* supernatant bound erythrocytes. This represents an approximately 70% reduction in overall PY235 binding in the *PYΔpy01365(NF2)* parasite clone and could explain the invasion properties observed. In contrast there appears to be very little difference between YM and *PYΔpy01365(NF1*) with 27% of total mcAb 25.77 reactive PY235 of YM and 28% of *PYΔpy01365(NF1*) being able to bind to erythrocytes (Fig. 5B).

## Discussion

Previous studies looking at the transcriptional profile of *Py235* in *P. yoelii* have shown that different members of *Py235* are transcribed at different levels, with *PY01365* being transcribed at the highest level in both virulent and avirulent parasite lines [27]. This led to the proposal that merozoites expressing different members of Py235 display differences in their ability to invade rbc, leading to a population of parasites where merozoites that express the most potent member of Py235 are more abundant than those that express (a less efficient) Py235 member. Such a mechanism would give the parasite the ability to rapidly adapt to changes in the host cell environment but also to escape host immunity targeting the most abundant member of Py235. PY01365 in addition to being the most abundantly expressed member of the *Py235* multigene family has also been shown to be recognized by the protective monoclonal antibody 25.77 [41]. Based on these findings we genetically disrupted *py01365* and assessed its overall impact on parasite virulence and *Py235* expression levels.

The successful disruption of *PY01365* leads to a compensatory change in the transcription of the other *py235* members, though it is clear that the transcriptional changes are not predetermined but can be different in the two knockout lines generated. The fact though that the *py235* transcription patterns once set are stable in multiple infections would suggest that the transcription pattern is determined early during the generation of the knockout parasite and then is stable inherited. This is somewhat analogous to the expression patterns of the PfRH family were genetic disruption tends to be required to induce a change in the utilization pattern of PfRH.

Disruption of *PY01365* results in a delay in the development of parasitaemia during the early stages of the infection with *PYΔpy01365* parasites showing increased host cell selectivity as reflected by the increase in the SI. Infections initiated with the same amount of PY*Δ*py01365(NF1) or PY*Δ*py01365(NF2) resulted in a delay in host death as compared to YM. It is interesting to note that in PY*Δ*py01365(NF2) peak parasitaemia is restricted to about 40% on day 5 with parasite levels subsequently dropping to as low as 22% on day 9 before again gradually rising leading to the death of all animals by day 22. In contrast PY*Δ*py01365(NF1) reach a parasitaemia of ∼25% by day 6 and continue to rise to the same levels as YM by day 8 with all animals succumbing to the infection by day 9. These results suggest that *PY01365* plays an important role during the early stages of the infection and indicate that the ability of YM to invade a wider repertoire of rbc is in part due to the expression of *PY01365*, and this is supported by the increased SI of the knockout parasites. The difference in the length of survival observed in the two knockout lines is consistent with differences in *py235* expression impacting on the ability of the parasite to efficiently invade a wider range of erythrocytes. It is clear that after the initial delay during the first few days of the infection *PYΔpy01365(NF1*) shows no erythrocyte restriction and invades all erythrocytes with similar efficiency than YM. In contrast *PYΔpy01365(NF2*) appears to be restricted to about 40% of the circulating erythrocyte population and can only expand overall parasitaemia during the latter stages of the infection when there is an influx of young erythrocytes.

Py235 has been shown to directly bind to rbc and the ability of the merozoite to recognize and invade a rbc is dependent on the amount of Py235 as well as its corresponding receptor available to form an interaction [3], [45], [46]. In addition the binding strength of a specific Py235 with its receptor will directly influence invasion efficiency. The reduced ability of *PYΔpy01365(NF2*) to invade erythrocytes of all ages may be explained by the observation that while the overall levels of mcAb 25.77 reactive PY235 are similar to those observed in YM, there is an approximately 3 fold reduction in the amount of PY235 binding to erythrocytes (Fig. 5A), in contrast no difference in the overall binding of mcAb 25.77 reactive PY235 is seen in *PYΔpy01365(NF1*) (Fig. 5B). This reduced binding in *PYΔpy01365(NF2*) will directly impact on invasion efficiency and would be expected to restrict the erythrocyte subset suitable for invasion.

The observation that the protective monoclonal antibody 25.77 which had recently been suggested to recognize Py01365 is still able to recognize about 30–40% of schizonts in the knockout parasites by immunofluorescence microscopy shows that this antibody is able to recognize other members of Py235 as well. This is supported by the work from Ogun et al (submitted) which identified other members of PY235 that are recognized by the protective monoclonal antibody 25.77 in a *PY01365* knockout parasite. Considering the relatively high sequence conservation between the different Py235 this is not necessarily surprising. It does though suggest that it is feasible to functionally target multiple members of a multigene family with specific antibodies at the same time and this might be important to consider in any vaccination strategies targeting the *P. falciparum* or *P. vivax* RH members.

Our work here has indicated Py235 as a key factor important for reticulocyte invasion during the early stages of an infection. Importantly, the ability of this Py235 to mediate virulence requires high levels of expression and is dependent on the aberrant location of PyEBL to the dense granules. Furthermore, our work sheds new light on the factors that contribute to parasite virulence. The findings here also have important implications for our understanding of invasion efficiency and virulence in *P. falciparum.*

## Materials and Methods

### Parasite preparation

Male BALB/c mice of 6–8 weeks old were obtained from Sembawang Laboratory Animal Center, National University of Singapore, and subsequently bred under specific pathogen free (SPF) condition at Nanyang Technological University Animal Holding Unit. Mice were infected with cryopreserved stocks of *Plasmodium yoelii* YM strain by intraperitoneal injections and parasitemia was monitored by thin blood smears stained with Giemsa as previously described [47].

### Isolation of Schizonts

When the parasitaemia reached 40%–60%, mice were terminated and infected blood was collected by cardiac puncture with heparin (Sigma). Parasitized blood was washed once in incomplete RPMI 1640 (Invitrogen). Schizont stage parasites were separated and harvested using a 50%–80% Nycodenz (Sigma-Aldrich) gradient. Schizonts were cultured till maturity in complete RPMI1640 containing 20% FBS with gentle shaking at 37°C.

### Parasite Transfection

Matured YM schizonts were transfected with linearized construct b3D-py01365 using the Basic Parasite nucleofector solution kit II (Lonza) with Amaxa electroporator and the published protocols [48], [49]. Transfected parasites were then introduced into new BALB/c mice by intravenous injection and tranfectants were selected with pyrimethamine (Sigma) [50]. *PYΔpy01365* parasites were obtained by single-parasite cloning and integration was confirmed by PCR and Southern Blot (Fig. 1).

### Parasitaemia growth curve

To assess virulence, 5 mice as a group were injected intravenously with 10^4^ mature schizonts of either YM or *PYΔpy01365*, and parasitemias were monitored using thin blood films stained with Giemsa from day3 post-infection.

### Selective Index (SI) determination

Selectivity index as a parameter to determine the selectivity of a parasite to multiply invade a red blood cell has been previously described [27]. Selectivity index was calculated by dividing the observed number of multiple invasions over the expected number of multiple invasions in parasitized red blood cells. For SI determination, parasitaemias were counted using Giemsa stained thin blood films of 5%–15% parasitaemia [51]. Statistical significance of differences in SI was determined using Students *t*-test.

### Immunofluorescence Microscopy

Isolation of schizonts was carried out as described before. For Immunofluorecense assays, the schizont pellet was re-suspended in iRPMI and a small volume was used to make smears on glass slides. The smears were air dried, fixed for 1 min in ice-cold acetone and stored at −80°C. Slides were thawed, ice-cold acetone-fixed for 5 min, pre-incubated with 3% BSA at 37°C, then incubated either with mcAb 25.77 [8] alone for single labeling or mixed together with mcAb 25.77 and α2T8 [42] for double labeling at 37°C for 1 h. Later they were incubated either with Alexa fluor-594 conjugated goat anti-mouse IgG(H+L) alone for single labeling or mixed together with Alexa fluor-594 conjugated goat anti-mouse IgG(H+L) (Molecular Probes) for double labeling at room temperature for 1 h in dark. Parasite nuclei were stained with DAPI. Washes were done between two antibody incubations and after DAPI for 3X, 7 min with ice-cold 1X PBS. Slides were viewed under Olympus fluorescence microscope at 100X magnification by adding mounting medium for Fluorescence (Vector Laboratories, Burlingame, CA).

### Preparation of *P. yoelii* culture supernatant

Schizonts form both YM and *PYΔpy01365* were isolated and cultured in incomplete RPMI 1640 medium containing 20% fetal bovine serum (Invitrogen). Culture medium containing released soluble proteins was harvested after 16 h and supernatant was purified as previously described [43], [44], [52]. Briefly, the supernatant was harvested by two sequential centrifugation steps. The first one being carried out at 2100 rpm at 4°C for 3 min to pellet down the parasites followed by a second centrifugation step at 15,000 g at 4°C for 30 min to remove any remaining debris.

### Erythrocyte Binding Assay using *P. yoelii* culture supernatant

Erythrocyte binding assays were carried out as previously described [43], [44], [52]. The bound proteins were eluted and separated on a 6% polyacrylamide gel. Py235 was detected by Western blotting using mcAb 25.77 [8], [9] as previously described [52]. Quantification of Py235 in the supernatant as well as Py235 bound to erythrocytes was performed as previously described [52].

### cDNA preparation

Parasites were collected at day 4 or 5 after inoculation when parasitaemia levels were between 1–10%. Schizont pellets of either YM strain or *PYΔpy01365* were mixed with pre-warmed (37°C) Trizol LS (Invitrogen) with immediate vortex. RNA was extracted according to the Trizol LS protocol and then purified using RNA clean-up kit (Qiagen) according to the manufactured protocol. RNA quantitation was done using Nano-Drop. Residual DNA was removed by DNAse treatment using TURBO DNA-free™ kit (Applied Biosystems Inc), and cDNA was generated by superscript II reverse transcriptase (Invitrogen) as previously described [27].

### Real-time PCR

Unique primers were designed for 11 members of the py235 genes to amplify short regions ranging from 143 bp to 212 bp (Table 1). Genomic DNA extracted from YM infected blood using the Easy-DNA kit (Invitrogen) was used as an internal standard to compare the primer pair efficiency. cDNA was generated from at least three animals either infected with YM or *PYΔpy01365*. Both cDNA samples and genomic DNA samples were amplified with Sybr Green Master Mix (Applied Biosystems Inc) and analysed on ABI 7000 thermocycler. 18sRNA was used for normalization [15], [27]. Statistical significance of difference in transcript levels of the *py235* genes in the YM and KO was determined by Students *t*-test.

**Table 1:** Unique PCR primers used for Real Time-PCR.

Primer	Sequence	amplified fragment
PY03534F	AAACCCAAGTATAATGATAATAATAATG	4951–5131 → 181 bp
PY03534R	GATAGTGAGTACCATATTGTTTATATC	
PY03432F	TAACAAAATTTGTTAATACTATACGC	3733–3896 → 164 bp
PY03432R	GTTATTTTGGTTATCTATAACGATTG	
PY01365F	AAAAGATTAACTCAGGGCACGAATC	2576–2721 → 146 bp
PY01365R	CTCTTCAATGGATTTGGTTATTTC	
PY01185F	CACAACATGTAAATGATGTAAAATC	7307–7456 → 150 bp
PY01185R	GCATAGTATTAATGTATGCGTCTA	
PY02104F	CAATTTTAGAACCAGCAAAGTATG	5735–5915 → 181 bp
PY02104R	TTGTAATTAGTTTTTTCTGAAGATTTG	
PY05054F	TTATCGTTTGGTTCTCAAAATTATG	6071–6238 → 168 bp
PY05054R	GTAATCATTTTCTAATTGTTCGATAG	
PY06018F	TGATATTGATACATTAAACCAAAAAATC	1245–1397 → 153 bp
PY06018R	TTTGGATCCTCGTTAAACATTG	
PY03184F	AACAATTAAAAACCCTTGAGGAAC	4900–5042 → 143 bp
PY03184R	GTAATTCTTTTTATGCTGATTTACAG	
PY00649F	GACACTGAATTGTACAATATAAAGTC	901–1091→191 bp
PY00649R	GTACATTCGTCTTTGTAATTATCAGAT	
PY04930F	ACTAATAACAGTGATTATAACATCAAC	4382–4593 → 212 bp
PY04930R	GTTCTGAATCAATTTTCGTTTTATC	
PY04630F	AAGTAAGAGTTATAAAAAAAATATTTCTG	3873–4015 → 143 bp
PY04630R	CACTATTATGCTTTTGGGATTCTG	
PY05054F	GATAATATTTTAGAAGCATC	6082–6238 → 157 bp
PY05054R	AATCATTTTCTAATTGTTCGATAG	

### Ethics statement

This study was carried out in strict accordance with the recommendations of the NACLAR (National Advisory Committee for Laboratory Animal Research) guidelines under the Animal & Birds (Care and Use of Animals for Scientific Purposes) Rules of Singapore. The protocol was approved by the by the Institutional Animal Care and Use Committee (IACUC) of the Nanyang Technological University of Singapore (Approval number: ARF-SBS/NIE A002). All efforts were made to minimize the suffering.
